# Atmospheric pressure plasma treatment of skin: penetration into hair follicles

**DOI:** 10.1088/1361-6595/acef59

**Published:** 2023-08-30

**Authors:** Kseniia Konina, Theresa A Freeman, Mark J Kushner

**Affiliations:** 1 Nuclear Engineering and Radiological Sciences Department, University of Michigan, 2355 Bonisteel Blvd., Ann Arbor, MI 48109-2104, United States of America; 2 Thomas Jefferson University, Philadelphia, PA 19107, United States of America; 3 Electrical Engineering and Computer Science Department, University of Michigan, 1301 Beal Ave., Ann Arbor, MI 48109-2122, United States of America

**Keywords:** atmospheric pressure plasma, plasma treatment skin, modeling, plasma medicine

## Abstract

Sterilization of skin prior to surgery is challenged by the reservoir of bacteria that resides in hair follicles. Atmospheric pressure plasma jets (APPJs) have been proposed as a method to treat and deactivate these bacteria as atmospheric plasmas are able to penetrate into structures and crevices with dimensions similar to those found in hair follicles. In this paper, we discuss results from a computational investigation of an APPJ sustained in helium flowing into ambient air, and incident onto a layered dielectric similar to human skin in which there are idealized hair follicles. We found that, depending on the location of the follicle, the bulk ionization wave (IW) incident onto the skin, or the surface IW on the skin, are able to launch IWs into the follicle. The uniformity of treatment of the follicle depends on the location of the *first entry* of the plasma into the follicle on the top of the skin. Typically, only one side of the follicle is treated on for a given plasma pulse, with uniform treatment resulting from rastering the plasma jet across the follicle over many pulses. Plasma treatment of the follicle is sensitive to the angle of the follicle with respect to the skin, width of the follicle pocket, conductivity of the dermis and thickness of the underlying subcutaneous fat layer, the latter due to the change in capacitance of the tissue.

## 1. Introduction

Low-temperature, atmospheric pressure plasmas (APPs) are used in biomedical applications [1–3], including cancer treatment [4, 5] wound healing [6, 7] and sterilization [8, 9]. These beneficial interactions with human tissue are produced by a combination of electric fields, UV photons, and short and long-lived reactive species. One of the most common types of short- and long-term infections associated with healthcare are at surgical sites [10]. This complication dramatically increases mortality in high-risk groups [11]. To prevent these infections, prior to surgery sterilization of the skin in the vicinity of the incision is performed to minimize contamination of the surgical wound [12]. A confounding factor is that the skin surface is covered with pores that open into hair follicles which can harbor approximately 25% of the pathogens found on skin [13]. In addition to removing hair that may itself contain bacteria, the skin is shaved to better gain access to the interior of the follicles to treat the bacteria they may contain. Current pre-op skin sterilization techniques do not penetrate into these areas as most of the currently used disinfection agents are liquids, and due to the relatively narrow gap between the hair shaft and skin, the penetration of liquid disinfectants by the capillary effect is limited. This motivates alternate means of treating the in-follicle bacteria to eradicate this threat.

APPs have been proposed for sterilizing skin prior to surgery and for treating bacteria in follicles in particular [14]. The surface ionization wave (SIW) that forms when APPs interact with dielectric surfaces, such as skin, can propagate into narrow gaps that are commensurate or larger than the Debye length of the plasma in the SIW. Typical Debye lengths in atmospheric pressure plasma jets (APPJs) are from a few to a few tens of microns [15]. Plasma sterilization of skin and sterilization of follicles by APPJs is especially attractive for their having skin tolerable temperatures while delivering high fluxes of reactive oxygen and nitrogen species (RONS) [14, 16–19]. For example, it was demonstrated that plasma generated by the kINPen APPJ can penetrated into hair follicles (on unshaved skin) [20]. The mechanism of plasma penetration into hair follicles is sensitive to many patient and environmental factors.

**Figure 1.**
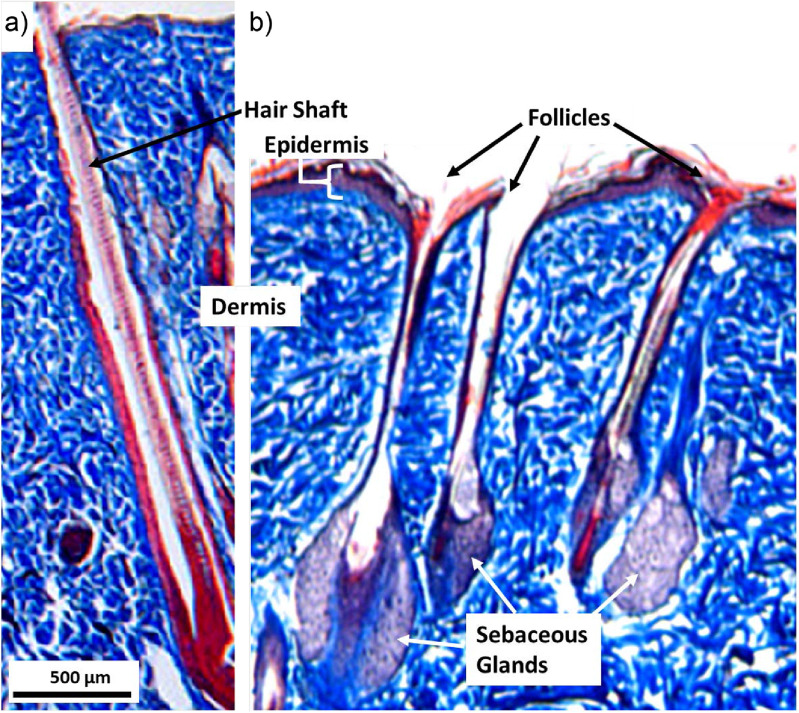
Trichrome histology of porcine skin with multiple follicles. (a) Typical follicle shows an example of the space that surrounds a prominent central hair shaft. (b) Multiple follicles with attached sebaceous glands. Some follicles lack a hair shaft.

A typical hair follicle (as shown in figure 1(a)) contains a hair shaft or strand (17–110 *μ*m diameter [21–23]) penetrating through the dermis and epidermis (skin) with a spacing between the shaft and surrounding tissue in the range pf 50–140 *μ*m. (This range of values is an estimate based on the total follicle diameter reported as 66–254 *μ*m [22, 24]). The gap between the hair shaft and skin varies depending on the individual and location on the body [22, 24]. The dimensions of gaps between hair shafts and skin, 50–140 *μ*m, suggest that APPJs may penetrate into the majority of hair follicles, and so have some ability to deactivate bacteria they may harbor. Finding treatment techniques that can penetrate into the follicle can also have added benefit in cancer treatment. Melanoma is one of the more aggressive types of skin cancer. Precursors of melanoma cells are thought to originate in hair follicles which then migrate to skin [25]. Melanoma has a high rate of recurrence [26], with some such risk being associated with ‘same spot’ recurrence, which may be follicles.

In this paper, we report on a computational investigation of the interaction of APPJs with idealized hair follicles in human skin. An APPJ sustained in He flowing into room air was directed onto a dielectric surface having the approximate electrical properties of human skin, punctuated with idealized hair follicles (after shaving). We found that plasma produced by APPJ can penetrate into typical follicle-like structures. The nature of propagation of plasma into the follicle is typically asymmetric. Given that our model is two-dimensional, the follicle is represented by a pair of capillaries separated by a hair shaft. Asymmetry refers to the plasma preferentially penetrating into one of the capillaries. However, even with asymmetric plasma penetration, photons and long-lived RONS appear to have a more uniform fluence on the inside surfaces of both capillaries. Short-lived RONS, important to interactions of plasma with human tissue and bacteria, typically follow the patterns of the plasma distribution inside the follicle. The orientation and angle of the follicle with respect to the SIW that the APPJ produced on the surface factor into the penetration and asymmetry of treatment. More uniform treatment of the follicles can be achieved with rastered jets. Patient and site specific factors are also important in determining the uniformity of treatment. For example, the conductivity of the skin (largely determined by oil and moisture content) affects the uniformity of plasma treatment of the follicle, as does the thickness of subcutaneous fat. The thickness of this fat layer varies from patient-to-patient and with location on the body for individuals. In our model, variation of mm to cm in the thickness of the fat layer produced significant variation in the propagation of plasma into follicles.

The model and methods that are used in this work are described in section 2. Results from the computational investigation are discussed section 3 for plasma interaction with idealized follicles for different follicle orientations and sizes. Our concluding remarks are in section 4.

## 2. Description of the model

This computational investigation was performed with the two-dimensional plasma-hydrodynamics model *nonPDPSIM* which is described in detail by Norberg *et al* [27]. *nonPDPSIM* is a modular simulator that uses time slicing techniques to solve neutral and charge particle transport, Poisson’s equation for electric potential and radiation transport on an unstructured mesh. Neutral gas flow is addressed by the integration of a compressible form of the Navier–Stokes equation reformulated for number density (as opposed to mass density). Charged particle transport is represented using Scharfetter–Gummel fluxes [28]. Continuity equations are based on a conservative finite volume discretization. Continuity equations for charged particle densities are implicitly integrated concurrently with Poisson’s equation for the electrical potential. Transport coefficients and rate coefficients for collisions of electrons are obtained from stationary solutions of Boltzmann’s equation for the electron energy distributions. The electron energy density conservation equation is updated to produce the electron temperature following every timestep of updating densities and potential. Time slicing occurs with the more slowly varying neutral flow field. Radiation transport is addressed with Green’s function approach. The photoionization model is based on emission of VUV radiation by excited states of helium He_2_^*^, He(3P), He(2^1^P) to ionize nitrogen molecules.

Following the discharge pulse, when there is no applied voltage, and after the plasma begins to dissipate, Poisson’s equation is no longer solved. We enforce charge neutrality in the afterglow, which then enables larger timesteps. During the discharge pulse, timesteps can be on the order of ps. In early afterglow (100 ns), the time step was 2 × 10^−11^s. The timestep gradually increases to 10^−7^s in the late afterglow.

The gas-phase reaction mechanism includes 42 species and 736 reactions. The following species are included in the mechanism: e, H, H*, H^+^, H^−^, H_2_, H_2_(r), H_2_(v), H_2_*, H_2_^+^, H_3_^+^, OH, OH^*^, OH^+^, OH^−^, H_2_O, H_2_O(v), H_2_O^+^, HO_2_, H_2_O_2_, H_3_O^+^, O_2_, O_2_(v), O_2_(r), O_2_^*^, O_2_^**^, O_2_^+^, O_2_^−^, O_4_^+^, O, O*, O^+^, O_3_, N_2_, N_2_(v), N_2_(r), N_2_^*^, N_2_^**^, N_2_^+^, N_3_^+^, N_4_^+^, N, N*, N^+^, He, He(2^3^S), He(2^1^S), He(2^1^P), He(2^3^P), He(3P), He(3S), He_2_^*^, He_2_^+^, He^+^, HeH^+^. A detailed discussion of the mechanism is in [29].

**Figure 2.**
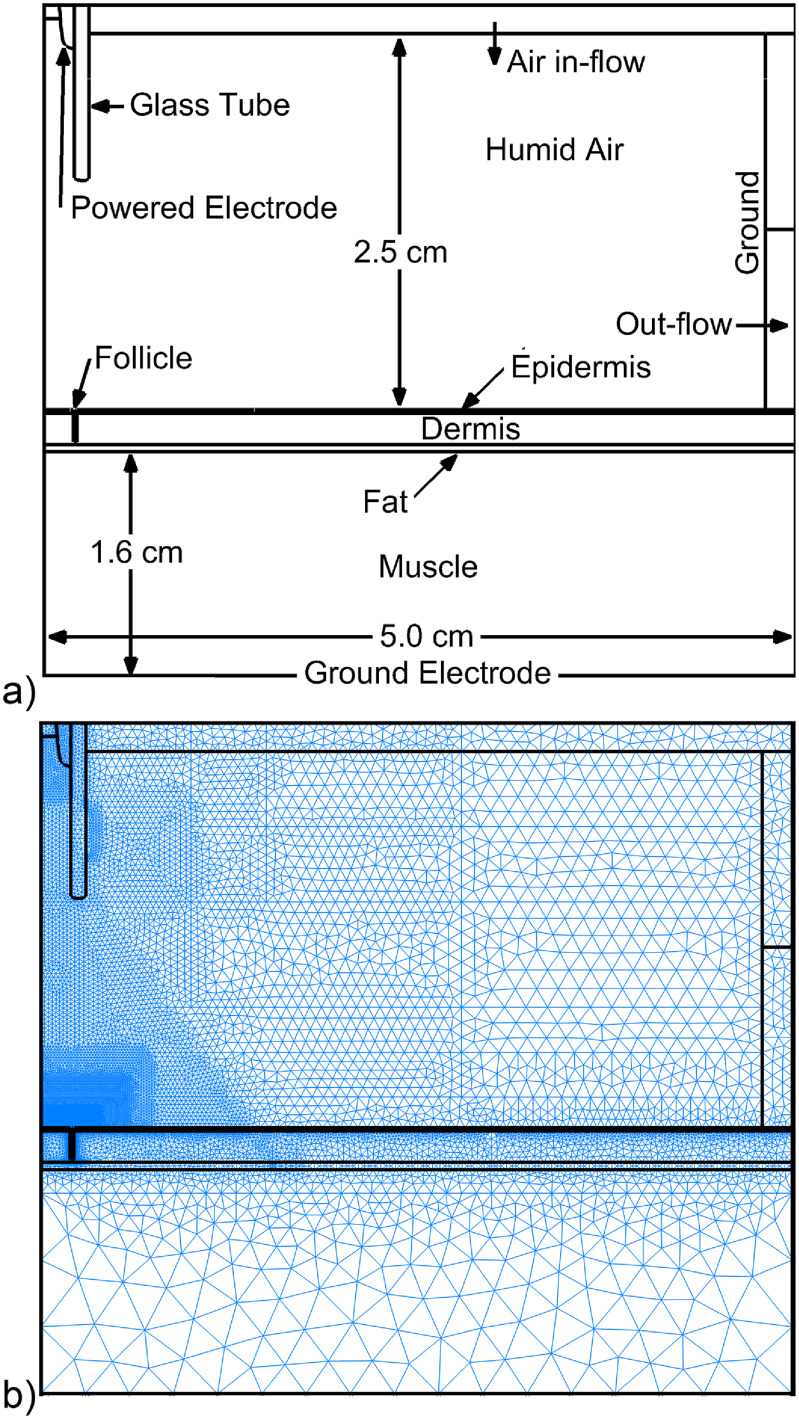
Computational domain. (a) Schematic of the system which is symmetric across the left boundary. (b) Numerical mesh.

A schematic of the Cartesian computational domain and numerical mesh are shown in figure 2. The computational domain is symmetric across the left border. The APPJ is produced by a ring powered electrode inside a glass tube (*ϵ*_r_ = 4) having an 4 mm inside width through which He is flowed at the rate of 1 lpm into an ambient of humid air (N_2_/O_2_/H_2_O = 0.795/0.2/0.005) at 1 atm. The He has air and water impurities (He/O_2_/N_2_/H_2_O = 1/2.4 × 10^−6^/4.7 × 10^−6^/10^−4^) and has a flow speed at the end of the tube of 1100 cm s^−1^. The end of the 1.2 cm long tube is 1.5 cm above the surface of the epidermis of the skin. The APPJ tube is surrounded at the top boundary by a nozzle through which ambient air is injected with an entry speed of 400 cm s^−1^. The purpose of the injected air is to account for entrainment of room air into the flow. All injected gases exit from the domain through the pump port on the right side that is also electrically grounded.

**Figure 3.**
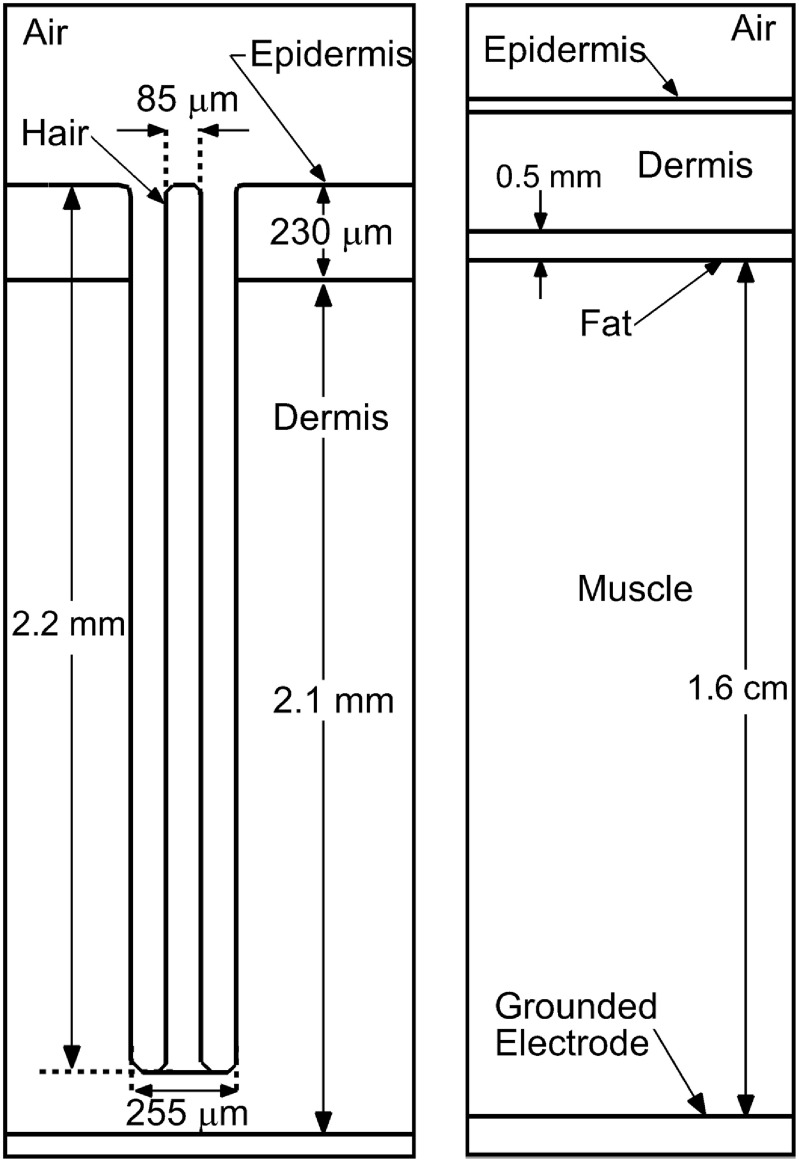
Schematic of the hair follicle and surrounding tissue used for the model.

The skin and hair follicle-like structure are represented by a set of dielectric materials, as shown in figures 2 and 3. The intent is to approximate treatment of a follicle on a human wrist with resolved epidermis, dermis, hair, fat and muscle. The dielectric constants were selected based on the material properties, adjusted so that capacitance of that particular component (e.g. muscle) resembled that of a wrist. The model skin has no conductivity in the base case and consists of a epidermis layer 230 *μ*m thick (dielectric constant *ϵ*_r_ = 10) over a dermis layer 2.1 mm thick (*ϵ*_r_ = 20). A 0.5 mm fat layer (*ϵ*_r_ = 5.3) sits between the dermis and a 1.5 cm thick muscle layer (*ϵ*_r_ = 15). The bottom of the muscle layer is grounded. The model hair follicle consists of a 255 *μ*m wide, 2.2 mm deep hole through the dermis and epidermis. A hair shaft (*ϵ*_r_ = 4.2), 85 *μ*m wide, is centered in the follicle hole. The straight edges of the follicle in the model are an approximation as actual follicles have curved edges and more complicated shapes, as shown in figure 1. The local environment around follicles is more complex due to the presence of blood vessels, glands and fluids or even the absence of a shaft as observed in some glandular follicles (figure 1(b)). The simplified geometry used here enables a focus on the fundamental properties of plasma penetration into follicles. On a case specific basis, more detail of the shape of the follicle and of its environment can be included.

The dielectric constants for skin and tissue were selected based on studies discussed in [30]. The high frequency dielectric constant for the hair shaft is based on its composition being primarily keratin [31].

The base case has a negligible conductivity for the dermis and epidermis. The motivation for this choice was to address short-lived SIWs on dry human skin whose pulse duration is short compared to the dielectric relaxation time. In section 3.4, we discuss the consequences of varying conductivity of the epidermis and dermis. The conductivity of the underlying muscle is not considered as the penetration of electric fields beyond the top few mm of tissue is small.

The choices of the thickness of the muscle and location of the ground plane were made based on approximating plasma treatment of a human wrist. The electrical representation and effective impedance of the human body, and location of the ground plane, are important considerations in use of any plasma medical device contacting the human body [32]. This sensitivity extends to *in vitro* studies where series capacitance and ground planes can affect effective dose [33]. For sufficiently short voltage pulses compared to dielectric relaxation times, this sensitivity decreases as the effective capacitance increases. We address some aspects of this sensitivity of treatment to the effective capacitance of the treated tissue through varying the thickness of the fat layer (section 3.4).

We acknowledge that a hair follicle penetrating through skin has many three-dimensional features, while this model is two-dimensional. The most glaring difference between a two-dimensional and three-dimensional representation is in the isolation of the two sides of the follicle in the two-dimensional representation. In 2D, plasma inside the follicle gap on the left side is isolated from the gap on the right side. In a 3D representation of a cylindrical hair shaft inside a cylindrical follicle, plasma could flow around the circumference of the gap from one side to the other. As such, our 2D representation is a worst-case analysis of plasma treatment of the follicle.

## 3. APPJ interactions with skin and follicles

**Figure 4.**
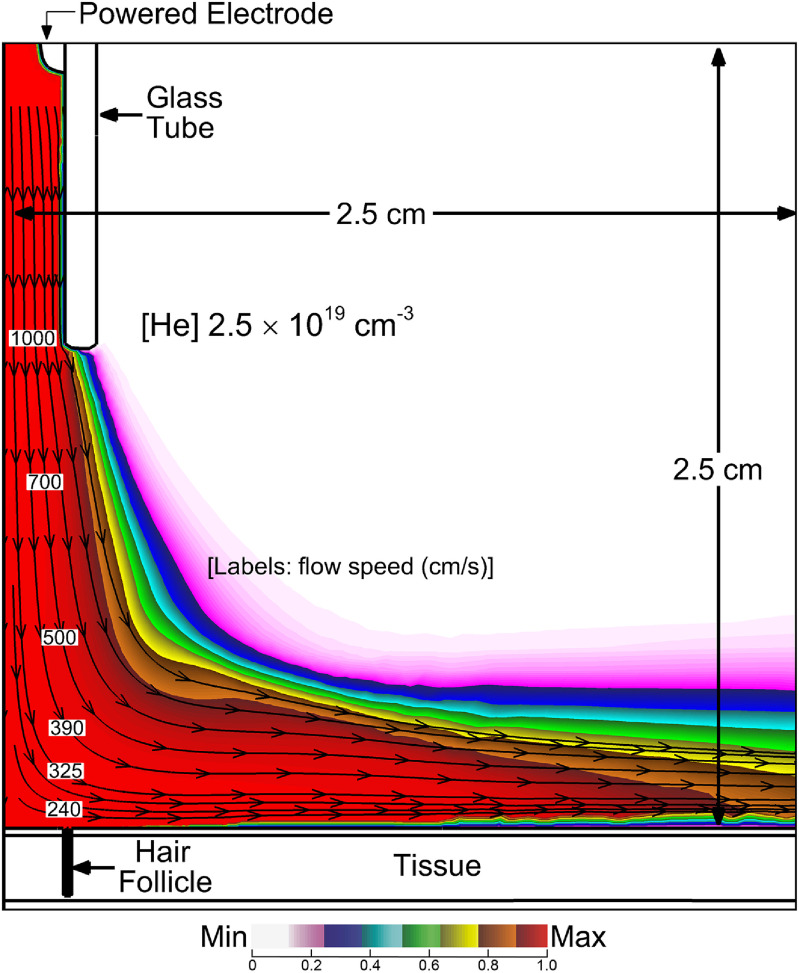
The stabilized concentration of helium before the plasma pulse with velocity streamlines. The streamline labels are speeds (cm s^−1^).

**Figure 5.**
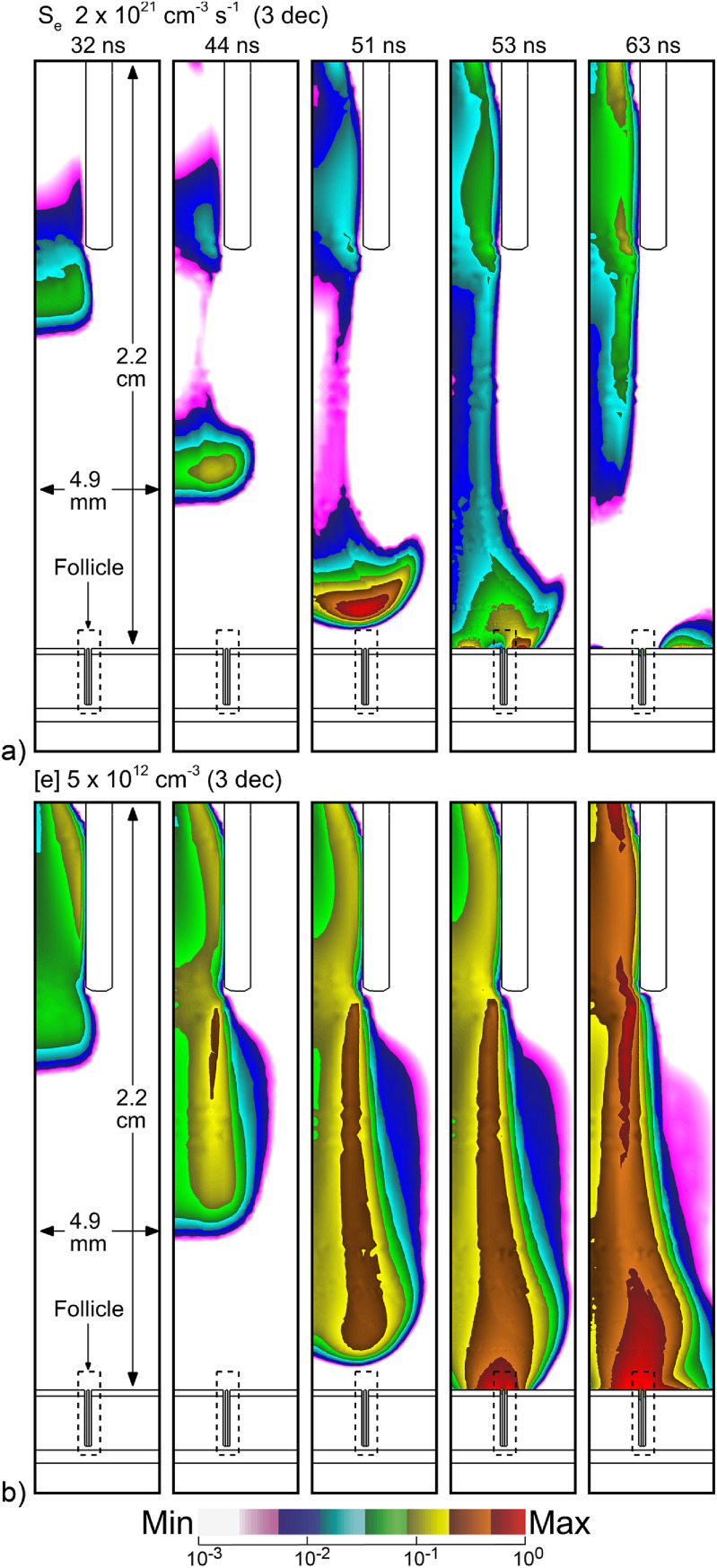
Plasma properties during propagation of the ionization wave onto the skin in the vicinity of the follicle. (a) Electron impact ionization source and (b) electron density. Images are plotted on a 3 decade logscale with the maximum value noted for each frame.

The base case for this investigation is the He APPJ treating the skin surface with a single hair follicle oriented perpendicular to the surface. Before applying voltage, fluid-only calculations are performed for 60 ms to obtain a stable gas flow field, with the results shown in figure 4. Following establishing the flow field, a voltage pulse of amplitude −25 kV is applied, having a total duration of 160 ns (step function rise, fall time 10 ns). The propagation of the ionization wave (IW) and resulting electron density are shown in figure 5. With the internal ring electrode, the IW initially propagates as an annulus, producing an annular distribution of electron density as observed experimentally with similar geometries [34, 35]. The IW crosses the 1.5 cm gap between the bottom of the tube and skin in about 23 ns, producing a streamer speed of 6.5 × 10^7^ cm s^−1^, commensurate with propagation speeds for similar conditions [35, 36]. As the IW approaches the skin, the rate of ionization intensifies to a maximum of 2 × 10^21^ cm^−3^s^−1^. The follicle was intentionally placed at approximately the location where the IW strikes the skin. That said, the electric field enhancement that occurs at the edges of the follicle intensifies the rate of ionization in the wave. When the IW strikes the skin, a reverse IW is launched back up the conductive plasma column. Coincidentally to the reverse IW, SIWs are launched in both directions along the skin (53 ns). The inward SIW dissipates while the outward SIW continues to propagate (63 ns). The resulting electron distribution is annular centered over the follicle, with a maximum density of 5 × 10^12^ cm^−3^.

**Figure 6.**
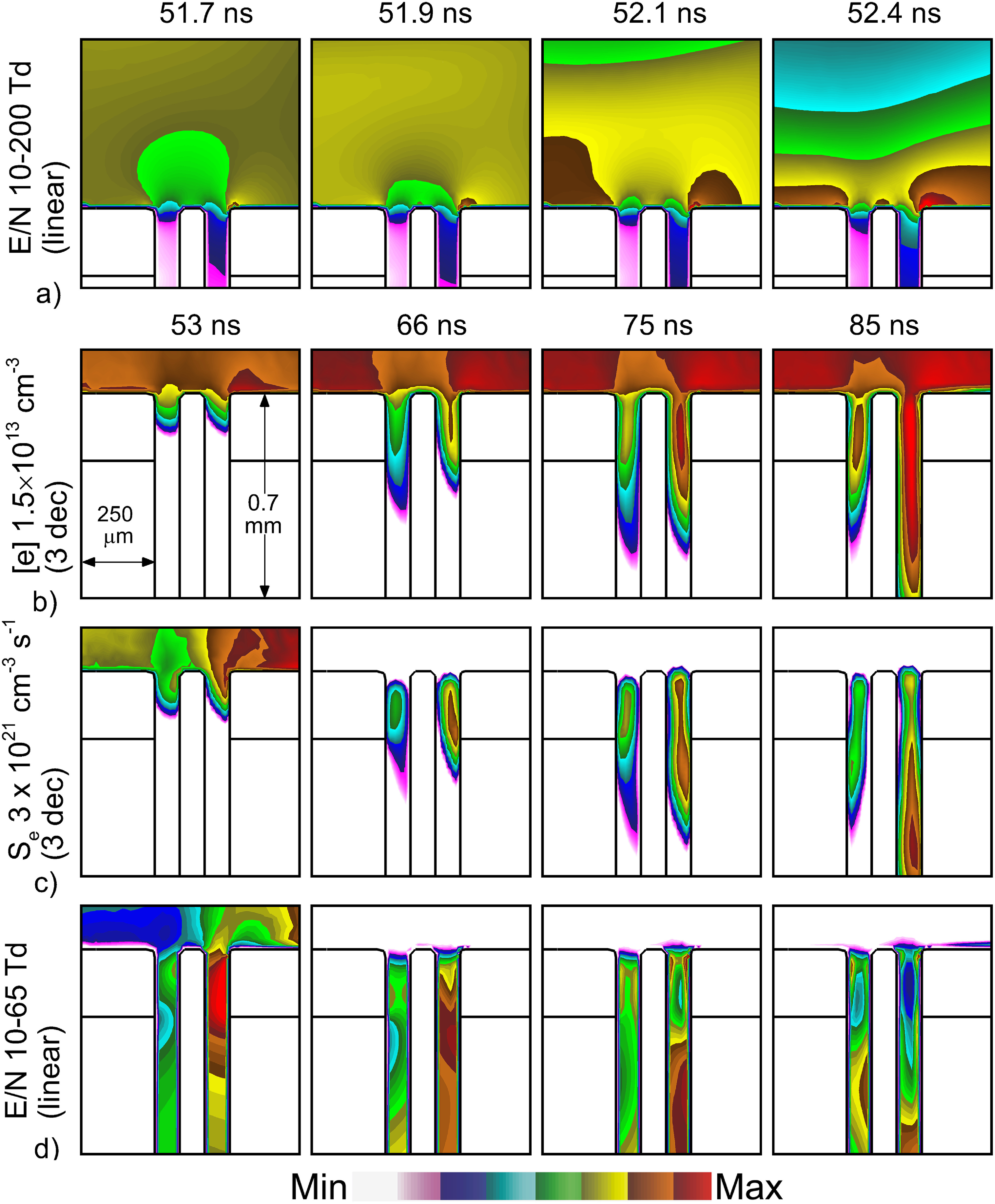
Propagation of plasma into hair follicles for the base case for times *t* = 51.7–52.4 ns. (a) *E*/*N* before plasma entry into the follicle (linear), (b) electron density (log-scale), (c) electron density (log-scale) and (d) *E*/*N* (linear). The logscale images are plotted over 3 decades. Maximum values are noted for each frame.

**Figure 7.**
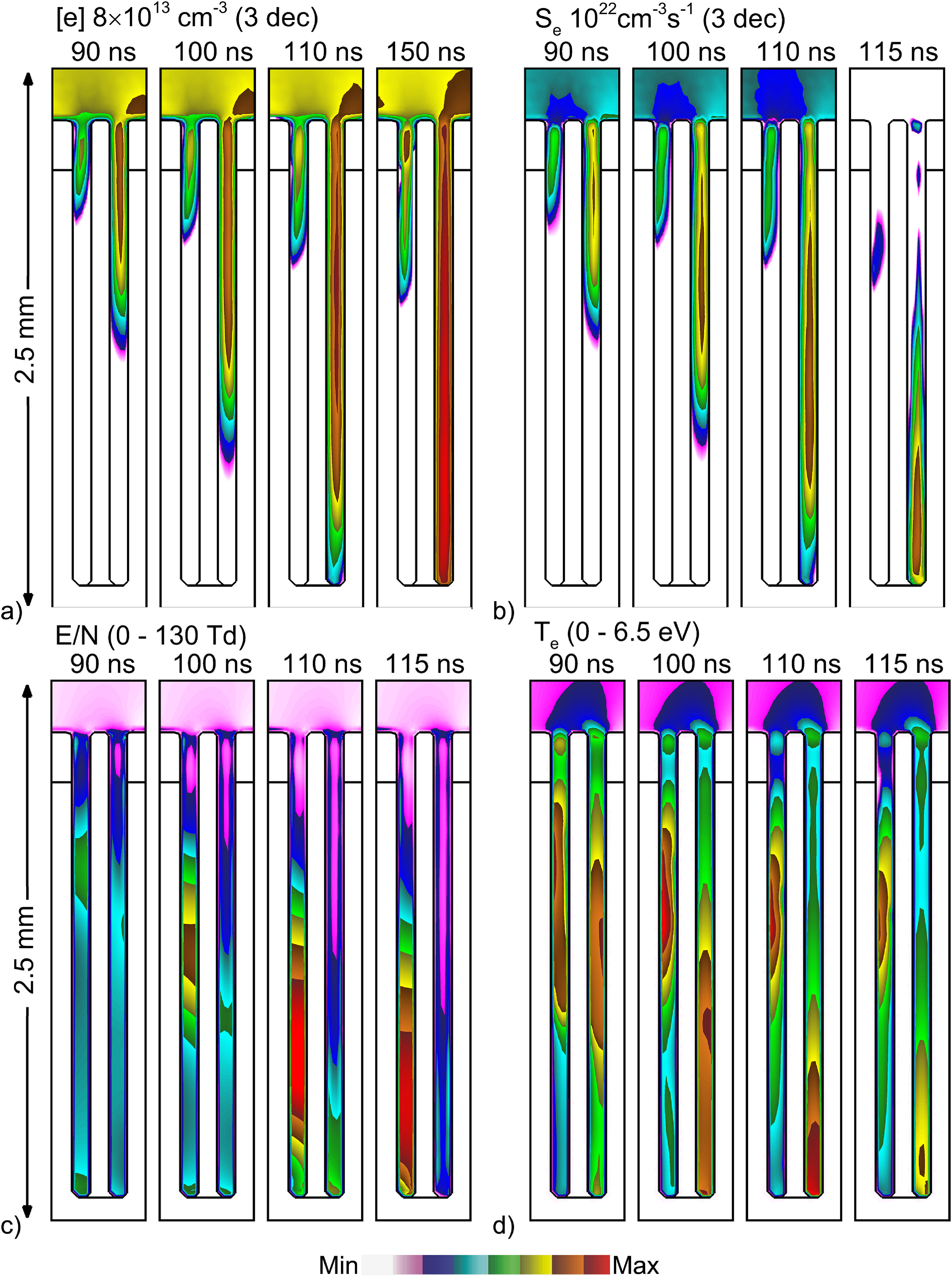
Deeper propagation of plasma into hair follicles for the base case for *t* = 90–115 ns. (a) Electron density (logscale), (b) electron impact ionization source (log-scale), (c) *E*/*N* (linear) and (d) electron temperature. The log-scale images are plotted over 3 decades. Maximum values are noted for each frame.

The electron density, electron impact ionization source and *E*/*N* (electric field/gas number density) are shown in figure 6 at the top of the follicle. The top row of images shows *E*/*N* prior to and just as the IW strikes the skin. The following images show the initial development of IWs and plasma in the follicle. Deeper propagation of IWs into the follicles (electron impact ionization source and electron density) are shown in figure 7. Note from figure 5 that the SIW along the top surface of the skin leaves the vicinity of the follicle by 55 ns.

As the IW approaches the skin and the location of the follicle, the electric potential is compressed in front of the IW due to the trailing conductive streamer column [37]. Due to the high permittivity of the epidermis (*ϵ*_r_ = 10), dermis (*ϵ*_r_ = 20) and hair shaft (*ϵ*_r_ = 4.2), polarization of these materials produce larger electric fields at the surface of skin than in the gap within the follicle. At 51.7 ns, *E*/*N* are 125 Td (1 Td = 10^−17^ V cm^2^) above the skin and 75 Td in the gap. As the IW wave approaches the skin, the curvature of the high permittivity epidermis and hair shaft produce electric field enhancement. *E*/*N* is 170 Td and 200 Td on the left and right edges of the follicle, and 150 Td on the hair shaft. This local electric field enhancement then sets the initial conditions for plasma propagation into the follicle.

Due to the particular location of the follicle, the trajectory of the bulk IW and other environmental factors that shape the electric field, the electron impact ionization source, *S*_e_, is marginally larger on the right side of the follicle than the left side. With electric field enhancement occurring at the top of the follicle, SIWs are launched into both sides of the follicle. However, with *S*_e_ being marginally larger on the right side, *first-entry* into the structure and first launching of the SIW into the follicle occurs in the right pocket. The term pockets refers to the space between the hair shaft and skin inside the follicle. The outer side of the pocket is skin, while in the inner side is the hair shaft. The larger enhancement of the electric field on the right side of the epidermis first launches an IW into the right pocket prior to launching the IW into the left pocket. The propagation of the IW in the right pocket is dominantly as a SIW on the outside skin surface, as this surface is charged negatively while the inner surface is not. The SIW is sustained by electron temperatures at the head of the SIW of up to 6.5 eV, with *E*/*N* of up to 65 Td. The right pocket fills with electron density of up to 8 × 10^13^ cm^−3^. At the end of the voltage pulse the ionization source by Penning ionization of O_2_ is about half that by electron impact ionization.

The propagation of the *first-entry* IW into the right pocket occurs from about 55 ns to the termination of the voltage pulse at 160 ns. During this time, there are no ionization sources in the gas phase above the follicle. The propagation is self-contained within the feature. That said, this propagation is driven in part by the charging of the top surface of the skin which, similar to a dielectric barrier, reaches a significant fraction of the applied voltage, in this case −12.6 kV by the end of the voltage pulse.

During *first-entry* into the right pocket, the high conductivity plasma of the right pocket affects the electric field in the left pocket. First, the high conductivity plasma in the right pocket shorts out the electric potential at that height which reduces *E*/*N* in the left pocket. Second, charging of the surfaces of the right pocket orients the electric field horizontally with electron acceleration to the left. The SIW hugs the outer right wall in the right pocket. Propagation of the SIW in the left pocket hugs the left wall. Later during the voltage pulse (90–150 ns), propagation of the IW stalls in the left pocket in spite of there being plasma in the channel at the top and significant *E*/*N* deeper in the channel. The lack of seed electrons deep in the channel and the electric field being oriented in an inopportune direction prevents propagation of the IW in the left pocket.

**Figure 8.**
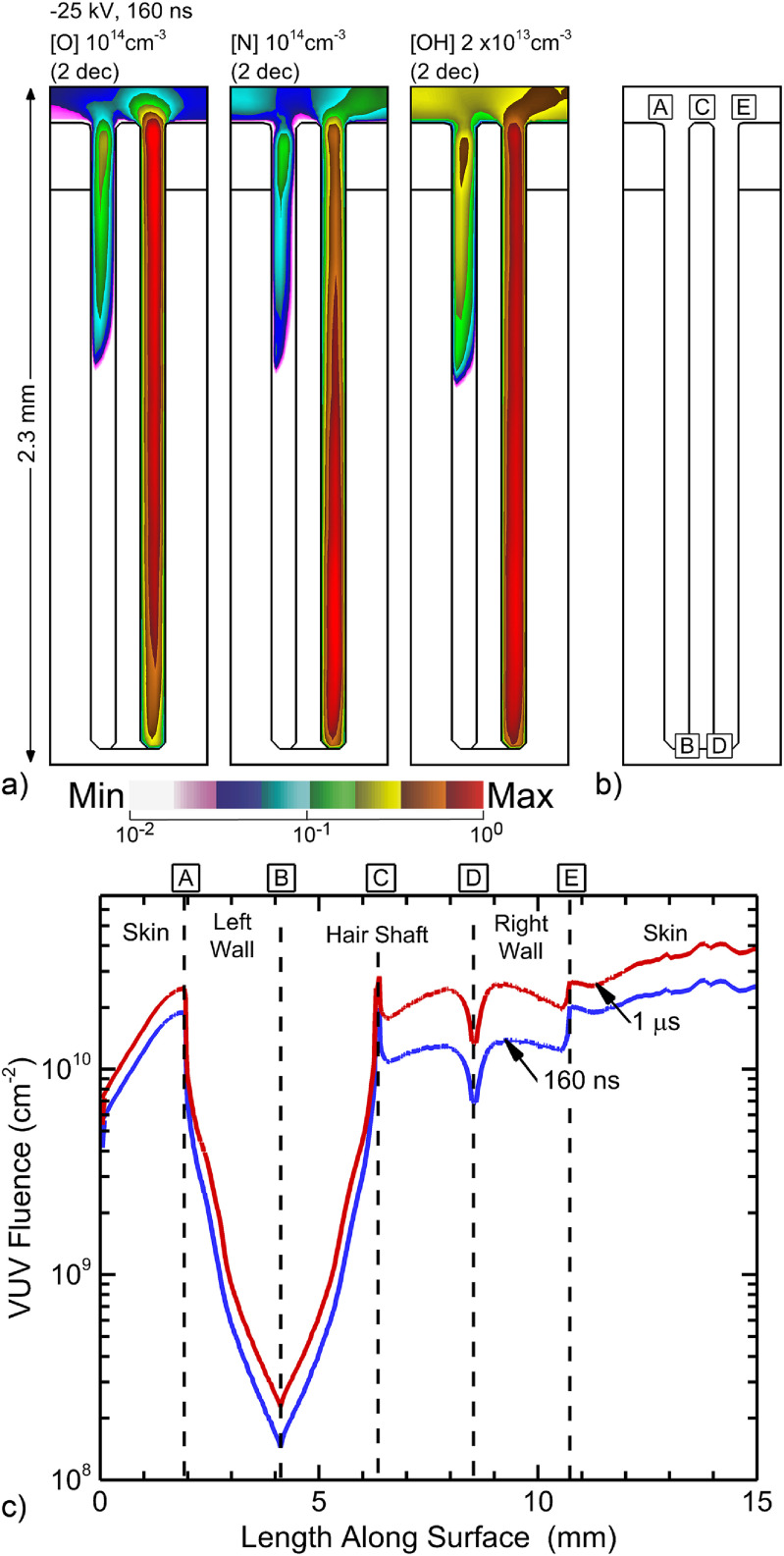
Radical and photon properties in the follicle. (a) Densities of O, N and OH at the end of the voltage pulse at 160 ns. Images are plotted on a 2 decade log-scale with the maximum value noted in each frame. (b) Reference locations for photon fluences. (c) VUV photon fluences to the inside surfaces of the follicle at 160 ns (end of voltage pulse) and 1 *μ*s. The locations along the surface of the follicle are indicated by the boxed letters.

Ultimately, sterilization of bacteria harbored in the follicle is a function of production of RONS and UV/VUV radiation. The concentrations of O, N, OH are shown in figure 8 directly after the voltage pulse. These distributions appear to follow that of the electron density as during this short time, there has been little opportunity for transport of neutral species beyond the location they were produced. The absolute densities of all radicals exceed 10^12^ cm^−3^. It is unlikely that radicals formed outside follicle will contribute to bacteria inactivation inside the follicle. At atmospheric pressure, the decay rate of radicals is relatively high, providing little time for radicals to transport from the bulk plasma into the follicle. Radicals not produced locally in the channel would need to survive many interactions with the walls in the narrow channel to reach deep into the follicle. Finally, gas flow will advect radicals and longer live species away from the follicles after the discharge pulse, as discussed below. As with the electron density, the treatment of follicles by these short lived RONS is also non-uniform—right pocket compared to left pocket.

One measure of *plasma dose* to the surfaces of the skin and follicles is the VUV fluence (time integral of flux) onto the surfaces. This VUV fluence after 160 ns (end of the voltage pulse) and 1 *μ*s is shown in figure 8(c) for points along the surface beginning on the top skin on the left of the follicle and continuing to the top skin on the right side of the follicle. The 50% increase in fluence between 160 ns and 1 *μ*s is due to dissociative recombination populating high lying atomic states, which then radiate. For some locations the fluence in the left pocket is up to 50 times smaller and far less uniform than in the right pocket. The fluence to the surface in the left pocket largely reflects the view-angle of the surface to the photon source at the top of the channel. The fluences to the surfaces in the right pocket largely are the result of locally emitting species. The small peak in fluence at the top of the hair shaft is due to VUV produced in the bulk plasma.

Asymmetry of plasma exposure is not necessarily the only measure of follicle treatment. Electroporation due to large electric fields also contributes to pathogen treatment [3]. The filling of plasma in one part of the follicle produces, through surface charging, large electric fields in portions of the follicle that are not filled by plasma. These large electric fields could contribute to pathogen treatment.

**Figure 9.**
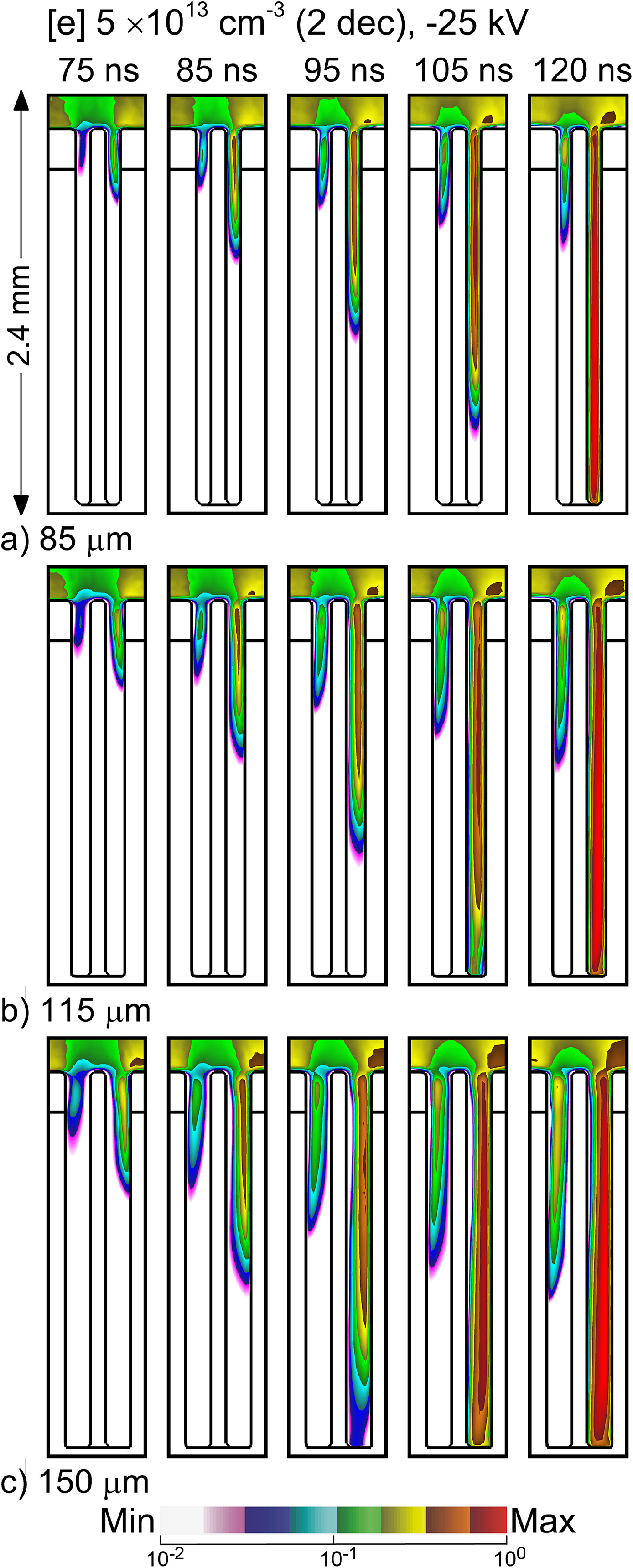
Time evolution of electron density inside hair follicles having different widths of pockets for *t* = 75–120 ns. (a) 85 *μ*m, (b) 115 *μ*m, and (c) 150 *μ*m. The images are plotted on a 2 decade log-scale having maximum value 5 × 10^13^ cm^−3^.

There is a large range in dimensions of hair follicles based on, for example, age and location on the body. The hair shaft itself varies in diameter from 17 *μ*m to 110 *μ*m, and the widths of the pockets can vary from 50 *μ*m to 140 *μ*m. The widths of the pockets in the follicle were varied between 85–150 *μ*m, while keeping the diameter of the hair shaft constant at 85 *μ*m to determine the consequences on plasma penetration on this variability. Electron densities in the follicle as a function of time and width of the pockets are shown in figure 9. A general trend is that the speed of propagation of the SIW in the right pocket increases with increasing width, with perhaps a transition to a bulk IW. With a pocket width of 85 *μ*m, the IW within the pocket propagates with a speed of approximately 5 × 10^6^ cm s^‒1^, with the speed being 8 × 10^6^ cm s^‒1^ for the pocket width of 150 *μ*m. With wider gaps charged particle losses to the walls are lower, which enables a higher propagation speed. For example, with the 85 *μ*m pocket, the IW charges both sides of the pocket (though dominantly the right side). With the 150 *μ*m pocket, the charging is dominantly on the right side, as the width of the pocket is commensurate with the width of the SIW.

In spite of the wider gap and more rapid filling of the right pocket, the left pocket largely remains unfilled with plasma. The penetration of plasma into the left pocket does improve with increasing width of the pocket, a consequence of more there being more isolation from the right pocket and more favorable entry of plasma into the pocket at the top of the follicle. At some width (and separation) the left and right pockets will be independent.

### 3.1. Orientation and location of the hair follicle

**Figure 10.**
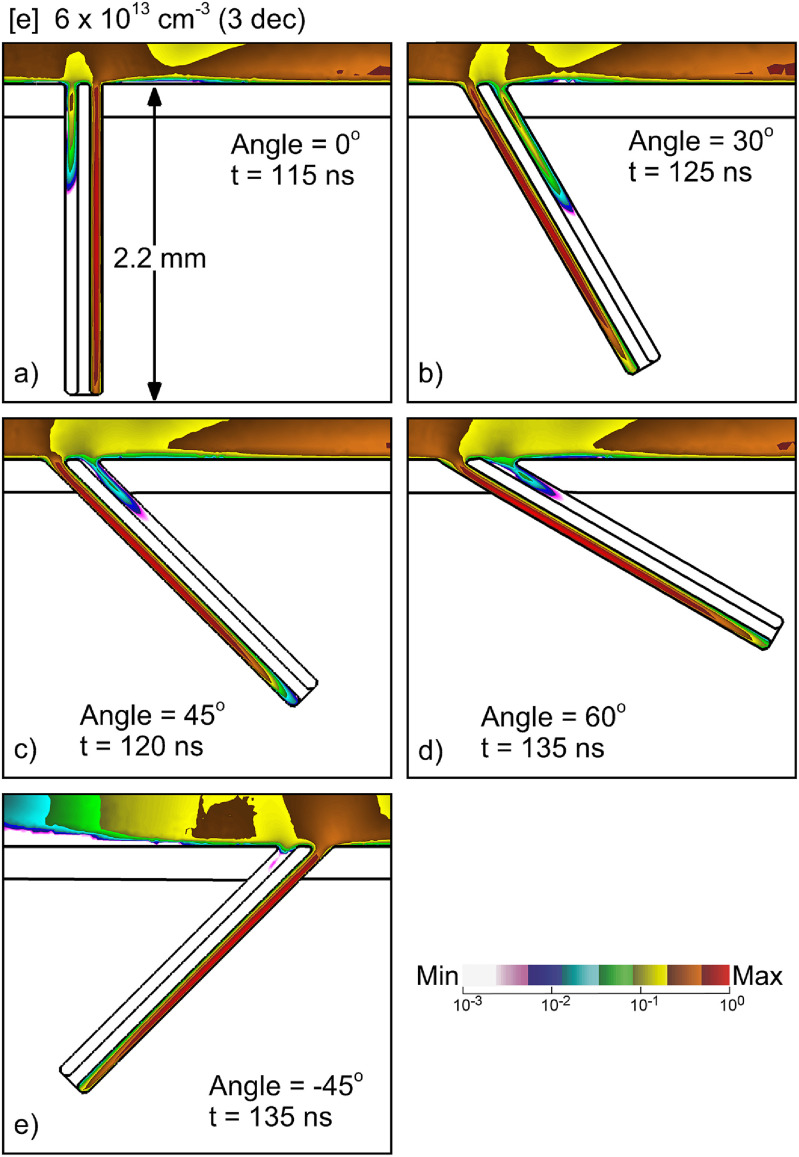
Electron density in the follicle for different angles with respect to the skin at the times the plasma reaches the bottom of the follicle. (a) 0° at 115 ns, (b) 30° at 125 ns, (c) 45° at 120 ns, (d) 60° at 135 ns and (e) −45° at 135 ns. The densities are plotted on a 3 decade log scale having maximum value 6 × 10^13^ cm^−3^.

Although hair follicles are generally oriented perpendicular to the skin surface, hair follicles may have random orientation with respect to the skin surface. The orientation of the follicle affects several critical factors for penetration of the plasma: curvature of the surface at the entry to the follicle, open area leading into the follicle and direction of the applied electric field. All factors may affect the *first-entry* of plasma into the follicle. Electron densities are shown in figure 10 for orientations of the follicle with respect to the skin surface from perpendicular (0°) to 60°, and a reverse direction for 45°. The position of the center of the hair shaft at the surface of the skin remains constant as the orientation is changed. All other parameters are kept the same as in the base case, besides the geometry of follicles. The time of each image is when the plasma column reaches the bottom of the pocket.

The orientation of the follicle does not have a large effect on their being plasma penetration into one of the pockets. The final plasma density and fill of one of the pockets is nearly the same for all orientations. However, the pocket that has *first-entry* and so fills with plasma is a function of orientation. With increasing slope, the pocket with *first-entry* and which fills with plasma changes from the right pocket to the left pocket. Since the SIW on the top skin surface propagates from left-to-right, the wider opening of the left follicle at the top surface at large angle enables a less inhibited entry of the plasma into the follicle. The left pocket then fills with plasma while the right pocket does not. With the follicle oriented at −45°, a larger area of the higher permittivity epidermis of the right pocket is exposed to the left-to-right SIW, and so the right pocket has *first-entry*, which then fills with plasma.

**Figure 11.**
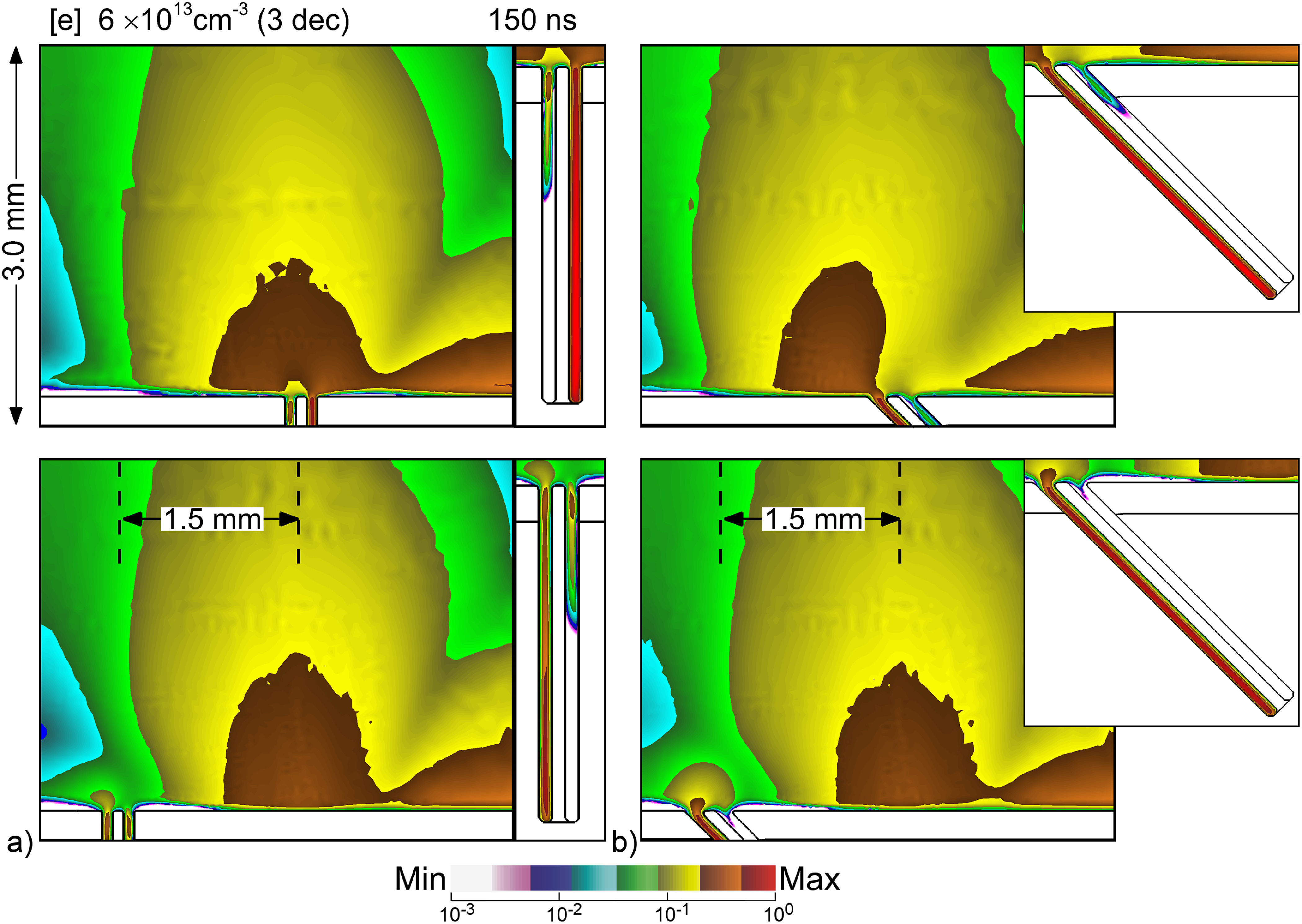
Electron density for different locations and angles of follicles at 150 ns. (a) Base case vertical follicle and a follicle shifted by 1.5 mm. (b) Follicle slanted at −45°and a slanted follicle shifted by 1.5 mm with the same geometry. The electron densities are plotted on a 3 decade log scale having maximum value 6 × 10^13^ cm^−3^.

Follicles that are oriented away from the perpendicular generally have less uniform of treatment, as measured by the plasma filling of left-and-right pockets. This non-uniformity is in large part a result of the increased likelihood that the SIW has for *first entry* on one side of the follicle as the orientation becomes further from the vertical. For example, a positive angular orientation (tilting to the right in figure 11) is likely to produce *first entry* on the left side of the follicle, while a negative angular orientation (tilting to the left) is likely to produce *first entry* on the right side. In the cases discussed to this point, the bulk IW striking the skin arrived at nearly the location of the follicle. However, statistically, the IW may strike the skin surface in different locations with respect to the location of the hair follicle, which then influences the *first entry* in the follicle. This will always be the case in treating real skin where hair follicles are generally randomly distributed. Since the permittivity of the epidermis, dermis and hair are large, the surface curvature of the follicle produces local electric field enhancement that can affect the arrival of the bulk IW at the surface of the skin. The location of a follicle with respect to the arrival of the bulk IW and the subsequent SIW is therefore likely to influence the *first-entry* and so the plasma propagation into the follicle.

Electron density is shown in figure 11 for two follicle locations separated by 1.5 mm and two slopes of the follicle (0° and 45°) with other conditions remaining the same. When transitioning the vertical follicle 1.5 mm to the left, the bulk IW strikes to the right of the follicle. With the subsequent SIW propagating largely to the right, there is not a SIW that propagates across the opening of the follicle. In this case, the polarization induced electric field enhancement occurring at the curvature at the top of the follicle provides the local ionization that enables *first-entry*. For this particular geometry, *first-entry* favors the left pocket. A similar trend occurs for the slanted follicle when shifted to the left. Polarization induced electric field enhancement produces local ionization above the follicle which then seeds *first-entry* into the left pocket.

The location of the follicle with respect to the bulk IW does feedback to the bulk plasma. Examine in figure 11 the bulk plasma up to 1 mm above the surface in the vicinity of the sloped follicle. Shifting the follicle has both qualitative and quantitative effects on the distribution of plasma above the surface.

The *first-entry* that determines which pocket fills with plasma is, to some degree, a stochastic process. *First-entry* depends on the location of the follicle with respect to the location where the bulk IW strikes the surface, the direction of propagation of the resulting SIW and the topology of the top of the follicle that determines electric field enhancement. In actual APPJ treatment of skin, the angle of the APPJ is not necessarily perpendicular to the skin. The orientation of the APPJ also likely plays a role. In actual treatment of skin with an APPJ, the jet is repetitively pulsed and rastered across the skin. Any given follicle is either on the right- or left-side of the APPJ and so the location of the follicle is not a critical parameter for uniform treatment. As the relative location of the follicle changes with rastering of the APPJ, it is likely that both pockets fill with plasma during the treatment.

### 3.2. Fluxes and densities in the afterglow

Treatment of the follicle, uniformity and magnitude, ultimately depends on the density and fluence (time integrated fluxes) of reactant species to the surfaces inside the follicle. With pulse durations of hundreds of ns and pulse repetition rates of hundreds of Hz to tens of kHz, the duration of the interpulse period (or afterglow) is much longer than the pulse duration. Although the plasma pulse determines the initial location and density of neutral RONS produced by electron impact, the transport and ultimate interaction with the tissue is determined by dynamics during the afterglow.

**Figure 12.**
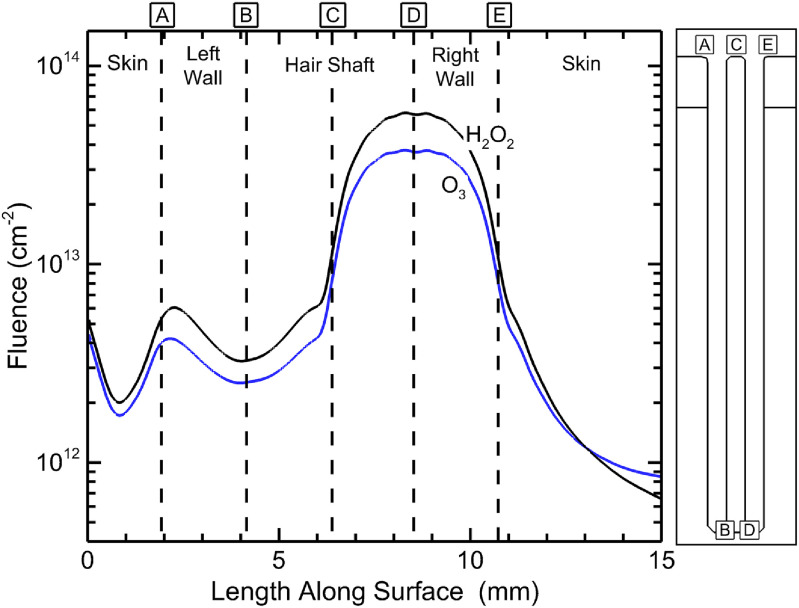
Fluence to surfaces inside the follicle of H_2_O_2_ and O_3_ at 1 ms in the afterglow. The schematic at the right indicates the locations along the inside surface of the follicle.

Short lived species, such as O and OH may have quite different initial distributions within the follicle, as shown in figure 8. Those short lived species generally have limited fluxes onto the surface as they can be converted to longer-lived species (O to ozone, O_3_ and OH to hydrogen peroxide, H_2_O_2_) prior to reaching a surface. For example, fluences of O_3_ and H_2_O_2_ to the inside surfaces of the follicle and the adjacent skin are shown in figure 12 after 1 ms of afterglow for the base case. The fluences to surfaces for the left pocket are focused towards the top of the follicle due to the limited penetration of plasma. The fluences to the surface in the right pocket are focused toward the bottom of the follicle. This trend results from the plasma being somewhat more intense in the bottom of the follicle. The OH density after the voltage pulse (figure 8) is 1.4 × 10^13^ cm^−3^ at the bottom of the follicle and 1.1 × 10^13^cm^−3^ at the top. However, the majority of the smaller fluence at the top of the follicle is due to transport of O_3_ and H_2_O_2_ out of the follicle during the afterglow.

The APPJ used in this investigation consists of a flow of largely helium initially perpendicular to the skin, which produces a stagnation point at the surface of the skin below the nozzle and flow outwards parallel to the skin. (See figure 4). The flow is parallel to the surface above the follicle with flow speeds above the follicle in the boundary layer of a few hundred cm/s. Inside the follicle, the advective flow is stagnant, with transport being diffusive. The production of O and OH, precursors to O_3_ and H_2_O_2_, occurs during the plasma pulse and immediate afterglow. Little additional O and OH are produced after about 200 ns. Conversion of these radicals to longer lived species (O_3_ and H_2_O_2_) occurs over the tens of microseconds to ms timescales.

**Figure 13.**
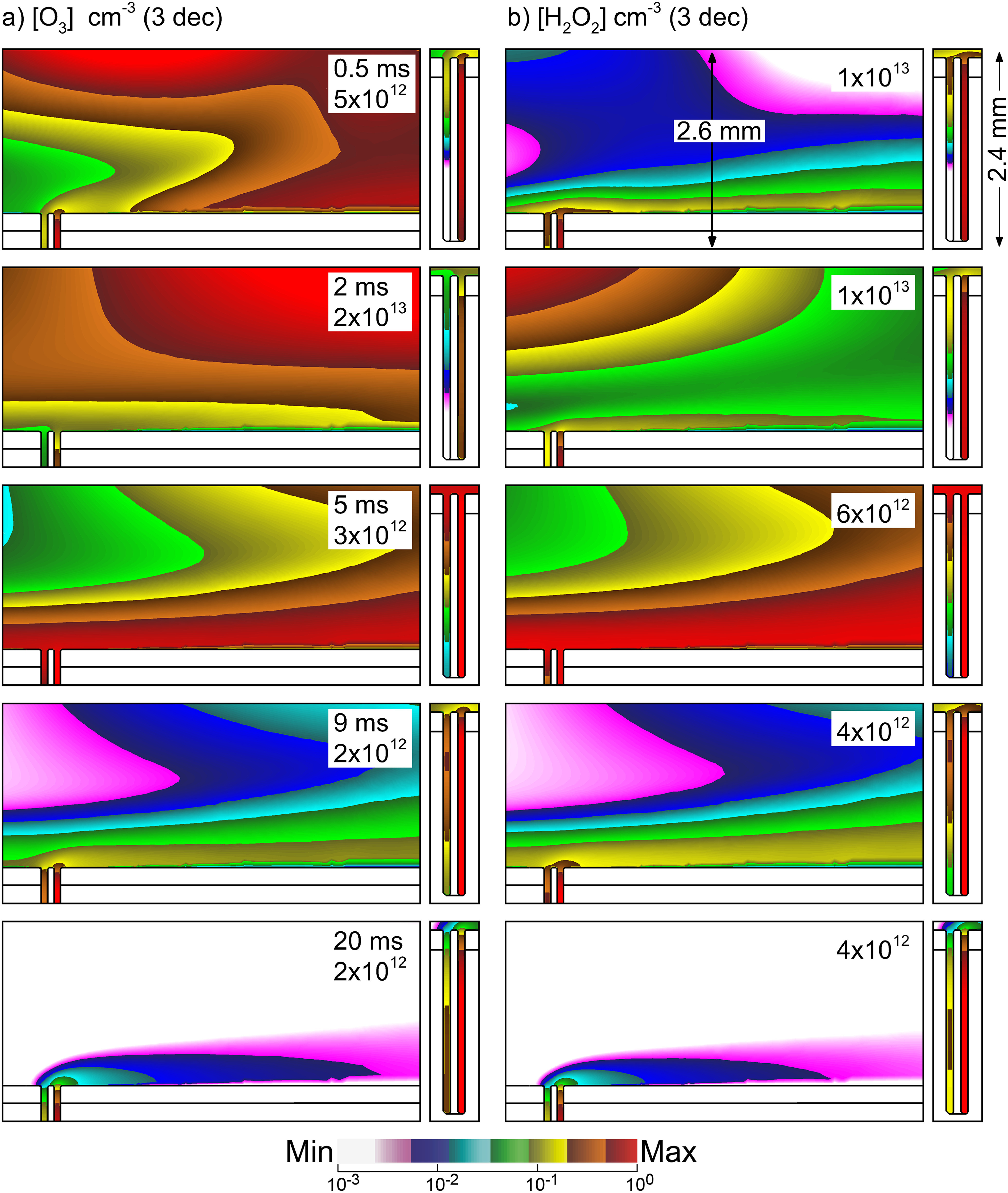
Time evolution of the densities of ROS inside the hair follicle and above the skin in the afterglow following a plasma pulse. (a) O_3_ and (b) H_2_O_2_. The densities are plotted on a 3 decade log-scale with the maximum value (cm^−3^) noted in each frame.

The densities of O_3_ and H_2_O_2_ during the post-plasma afterglow are shown in figure 13 for times up to 20 ms. With the bulk gas flow parallel to the surface during the afterglow, the O_3_ and H_2_O_2_ in the bulk plasma are advected away from the vicinity of the follicle until there are negligible densities of O_3_ and H_2_O_2_ above the follicle that originate in the bulk plasma. The O_3_ and H_2_O_2_ in the follicle are largely unaffected by the bulk gas flow. With there being little O_3_ and H_2_O_2_ in the bulk gas, the O_3_ and H_2_O_2_, in the follicle now have the largest densities. With the large aspect ratio of the follicle, the O_3_ and H_2_O_2_ slowly diffuse out of the follicle producing a plume that is advected downstream. In practice, the APPJs used for treatment of follicles will be rastered, which at times might produce advective gas flow in a direction that opposes the diffusion of reactants out of the follicle [38]. With typical diffusion times to empty the follicle of reactants being tens of ms, this situation might occur with rapid rastering. That said, these opposing flows would likely redistribute the reactants that diffuse out of the follicle over the adjacent skin as opposed to slowing or preventing the rate of diffusion out of the follicle.

### 3.3. Fat layer thickness

The thickness of the subcutaneous fat layer under the skin can significantly vary from person to person and from one part of the body to another. From an electrical perspective, the fat layer is a series capacitance for currents that return to ground through the body. Thin fat layers, corresponding to large capacitance provides a smaller impedance, and less voltage dissipation across the fat layer. Thick fat layers have small capacitance, larger impedance and more voltage dissipation. Since a thicker fat layer increases the distance between the powered electrode and the effective ground plane, the initial voltage available for plasma formation is reduced.

**Figure 14.**
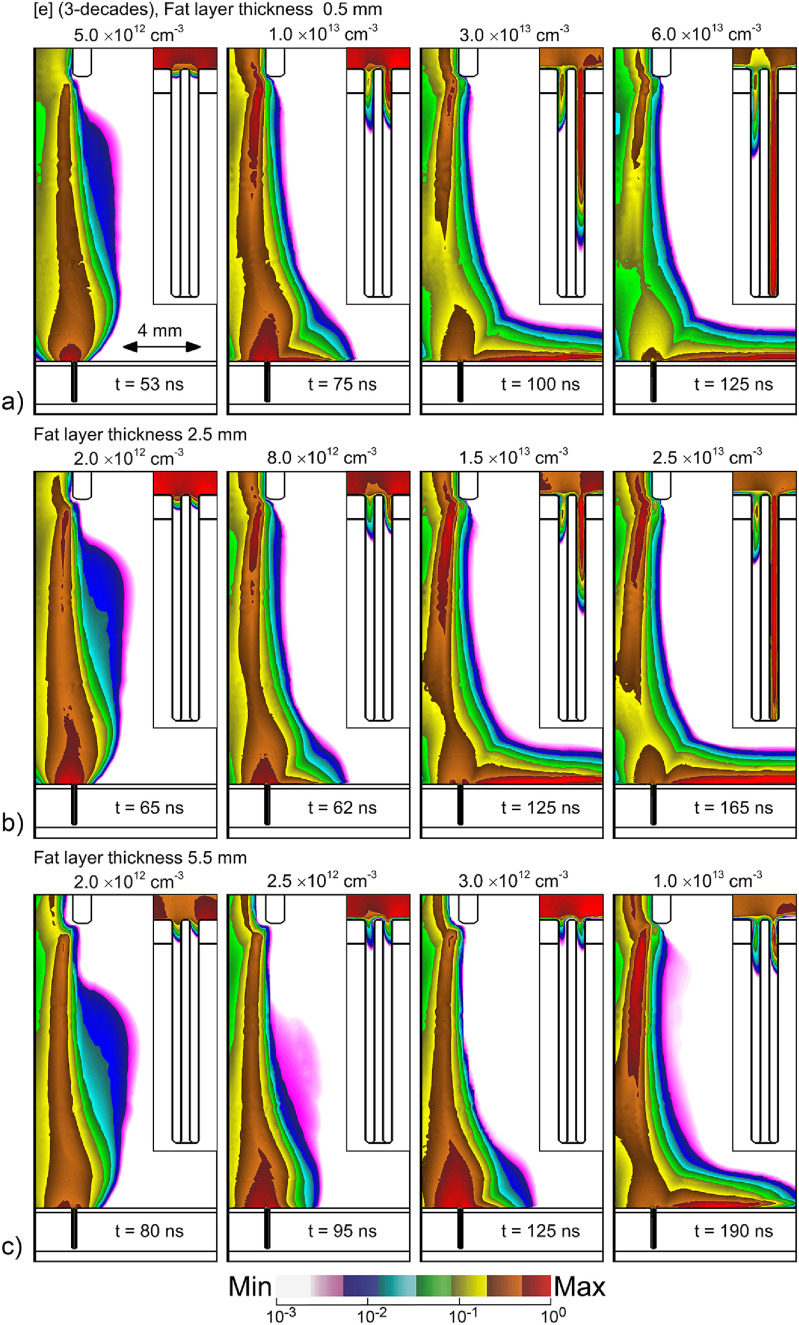
Electron densities in the nozzle-skin gap and inside the follicle at different times during and following the voltage pulse for different thickness of the fat layer. (a) 0.5 mm, (b) 2.5 mm and (c) 5.5 mm. The densities are plotted on a 3 decade log scale with the maximum value and time indicated in each frame.

The electron density is shown in figure 14 in the electrode-skin gap and in the follicles for fat layers of thickness 0.4–5.5 mm. With increasing thickness of the fat layer, the total capacitance in series with the plasma decreases. The decrease in capacitance has several repercussions. The first is that that with the large impedance of the fat layer, there is initially less voltage available for the gap, which produces a slower bulk IW. The time for first-touching of the bulk IW onto the skin (first column in figure 14) increases with increasing fat thickness and decreasing capacitance. The second effect is that the dwell time of the plasma over the follicles is longer with larger capacitance (thinner fat layers), which provides more opportunity for the plasma to enter into the follicle. The third is that the smaller capacitance (thicker fat layer) charges more rapidly, leaving less voltage available to launch the SIW into the follicle. The end result is that for otherwise the same conditions, there is less plasma penetration into follicles above thicker fat layers having smaller capacitance.

There are remedies that will produce plasma propagation into the follicles regardless of fat layer thickness—such as smaller gap between the jet and skin, higher voltage, rastering the jet. These results are intended to emphasize the APPJ will respond differently when treating different areas of the patient, and will respond differently patient-to-patient, due to the differences in the equivalent electrical circuit that the APPJ sees.

### 3.4. Skin properties

**Figure 15.**
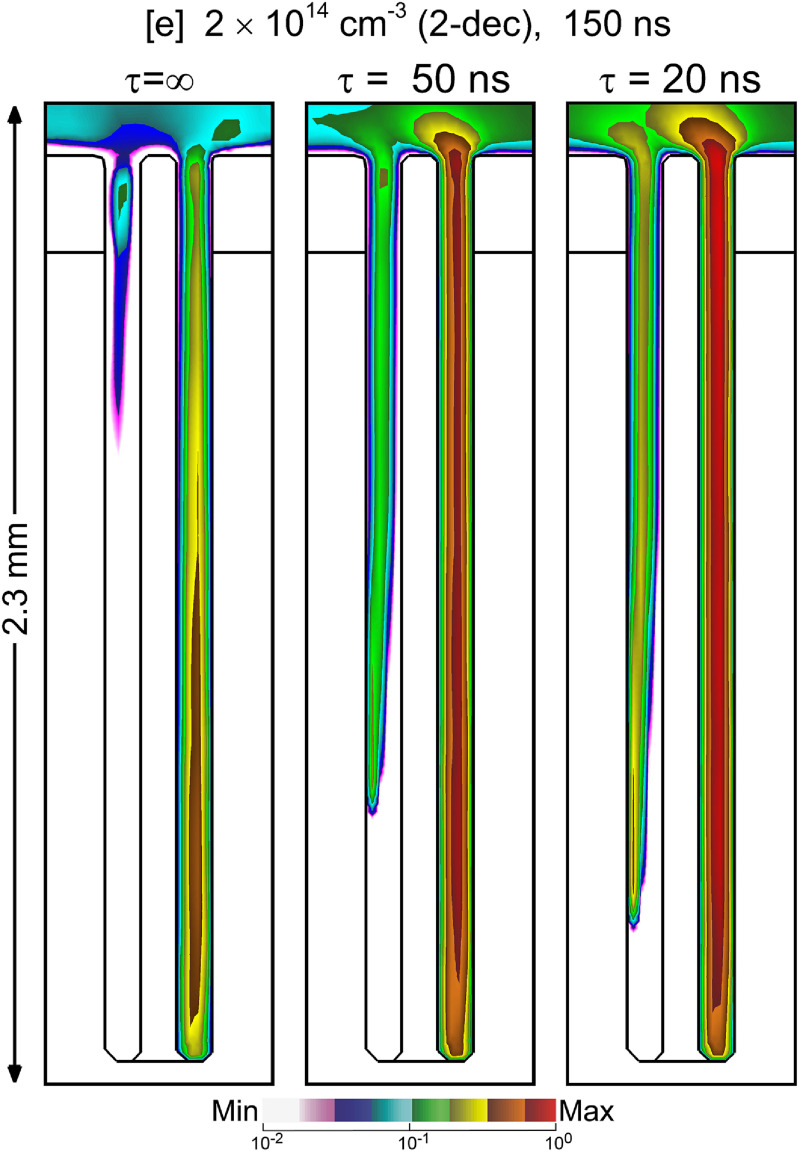
Election density at 150 ns for three skin conductivity and dielectric properties corresponding to dielectric relaxation times of infinite, 50 ns and 20 ns. The densities are plotted on a 2 decade log scale having maximum value 2 × 10^14^ cm^−3^.

Electrical representation of the skin and follicle in the model are as lossy dielectrics—materials with a dielectric constant, *ϵ*, and conductivity, *σ*. These values determine the dielectric relaxation time of the tissue, *τ* = *ϵ*/*σ*. These dielectric properties of skin can significantly vary from person to person, by body location (arm, leg or face), and even depend on respiration or activity level of a given individual. Specific skin structures, like sebaceous glands produce liquids that contain salt, or lipid molecules that will also effect skin conductivity [39]. The treatment of follicles in skin having different dielectric relaxation times was examined. While all base case parameters and geometry were kept the same, the conductivity of the epidermis and dermis was varied to yield dielectric relaxation times of infinite (pure dielectric), 50 ns and 20 ns. The resulting electron densities in the follicles at the end of the voltage pulse are shown in figure 15.

Increasing the conductivity of the skin layer generally improved the uniformity of plasma treatment of the follicles. A purely dielectric, non-conductive skin layer charges as would a capacitor or electrode covering material in a dielectric barrier discharge. As skin has a finite conductivity, some current can continue to flow when there is less surface charging. With less surface charging, electric fields retain a component downwards in the direction of ground and so enhance the propagation of the SIW into the pockets. Physiological limits aside, there is a limit to the improvement to uniformity that can be achieved with increasing conductivity. High aspect ratio channels or vias in conductive materials are nearly equipotential with there being no component of electric field parallel to the surface. Such conditions would not be conducive to sustaining IWs into the feature.

**Figure 16.**
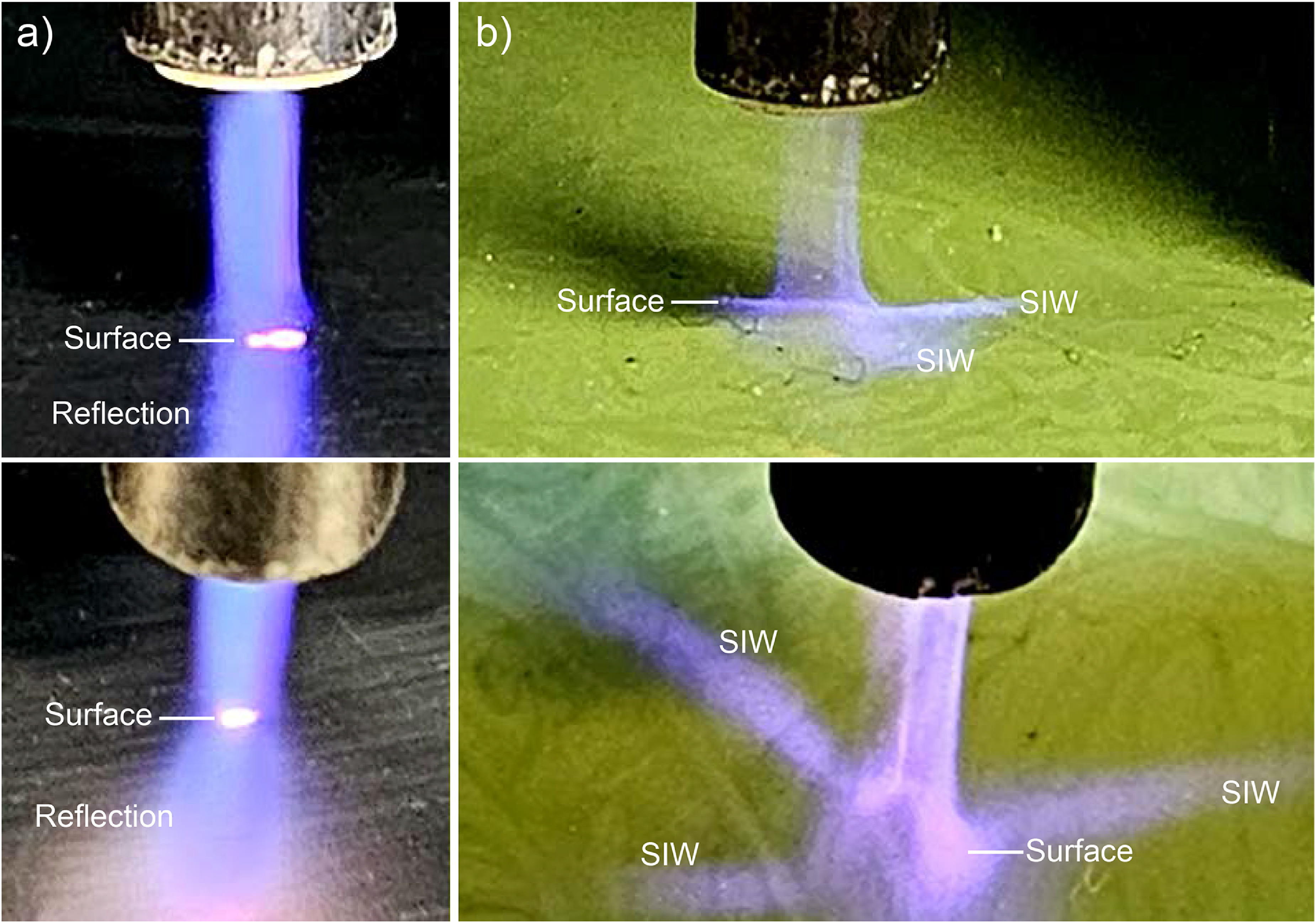
Conductivity of target substrate determines plasma flow properties. A helium plasma jet with a gas flow of 4 slm is shown. (a) Image of plasma with the target being a grounded metal plate. The top image is a side view. The bottom image is a view from approximately 45°. (b) The grounded metal plate is covered by a 3 mm silicon pad with the same distance between the plasma tube and surface. The propagation of surface ionization waves are noted.

The sensitivities to the dielectric properties of the surface of plasma jets striking surfaces are well acknowledged [40–42]. Given the large variability in skin electrical properties in moisture and oil content, and location on the body, these sensitivities should be a high priority in optimizing plasma treatment of follicles. An example of how combinations of capacitance and conductivity, and the ability of the plasma to contact ground, alters the plasma propagation along a surface is shown in figure 16 where a silicon pad is placed between an APPJ sustained in helium and the metal ground. While the images integrate over many plasma pulses and do not represent a single pulse, they illustrate the concept. When a conductive surface such as ground is immediately accessible, the spreading of the jet on the surface is minimal (figure 16(a) direct and lateral views). Lack of a lateral component of the electric field along the conductive surface inhibits spreading, and lack of charge accumulation on the surface preserves the voltage across the gap. The plasma jet is significantly less bright and spreads along the surface (figure 16(b)) using the same plasma conditions (power and gas flow) while placing a 3 mm silicon pad between the plasma and the ground. The propagation of SIWs are noted in the image. These phenomena, though less dramatic, will occur with significant changes in skin conductivity and affect propagation of the plasma into the follicles.

## 4. Concluding remarks

Sterilization of skin prior to surgery is challenged by killing of bacteria that is harbored in hair follicles. With a significant fraction of skin resident pathogens residing inside follicles and the limited penetration of liquid disinfectants, other forms of treatment of in-follicle bacteria are needed. From a structural perspective, hair follicles consist of high aspect ratio indentations in a mildly conducting, lossy dielectric—the skin. APPJs have the ability to penetrate into dielectric structures that have sizes commensurate to or larger than the plasma Debye length. A helium APPJ incident onto skin with idealized follicles was computationally investigated to determine the ability of plasma to penetrate into these structures, and their possible use for sterilization.

We found that there is a potential for non-uniform plasma treatment of the interior of hair follicles, at least with a single plasma pulse. The cause of that non-uniformity is that ionization waves that arrive onto the follicle, or SIWs that propagate to the follicle, have a *first-entry* point which establishes a local, high conductivity column having limited coverage within the follicle. The resulting conductivity of the plasma column reduces the ability of the plasma to propagate into the remainder of the follicle. The in-follicle coverage of plasma is a function of the width of the in-follicle gap between the hair shaft and skin, the angle of the follicle with respect to the top of the skin, the shape of the follicle opening which determines electric field enhancement, the conductivity of the skin and the location of the follicle relative to where the bulk ionization wave strikes the skin. To some degree, the point of *first-entry* of any given follicle is likely stochastic. Given that application of APPJs for skin sterilization will use high repetition rate pulses that are rastered across the surface of the skin, it is likely that uniform treatment of the majority of follicles can be achieved.

The precise reactive species that mitigate a given pathogen is an important consideration. Although short lived species may be quite non-uniformly produced in the follicle, diffusion of longer lived species such as O_3_ and H_2_O_2_ can produce significantly more uniform fluences to surfaces in the follicle.

The model that was used in this work is two-dimensional and represents a worst analysis of follicle treatment as there is not direct lateral transport of species between the left-and-right pockets. In actual follicles (cylindrical hair shaft inside a cylindrical cavity), transport around the shaft is possible and so long-lived species have an opportunity to diffuse throughout the follicle during the afterglow between pulses. With that said, our observations are best applied to those situations where short lived species and photons are the dominant biocidal agents.

Although we investigated hair follicles slanted at angles to the skin, hair follicles themselves are generally not straight channels. Typical follicles are narrow near the top and wider near the bottom, and have some curvature. Liquids are also present in the follicles along with gland entries. These structures may produce a non-uniform plasma inside the follicle but generally should not prevent plasma from penetrating.

Some follicles lack a hair shaft. A condition called alopecia represents a group of diseases resulting in hair loss [43]. It has been recently shown that there may be a benefit from plasma treatment of alopecia patients [44]. Follicles in alopecia patients tend to decrease in size but typically not drop below 130 *μ*m [45]. So the penetration of plasma into follicles likely occurs in alopecia patients, which may explain part of the benefit.

## Data Availability

The data that support the findings of this study are contained within the article and available from the corresponding author upon reasonable request.

## References

[1] Kong M G, Kroesen G, Morfill G, Nosenko T, Shimizu T, van Dijk J, Zimmermann J L (2009). New J. Phys..

[2] Adamovich I (2017). J. Phys. D: Appl. Phys..

[3] Gilmore B F, Flynn P B, O’Brien S, Hickok N, Freeman T, Bourke P (2018). Trends Biotechnol..

[4] Schlegel J, Köritzer J, Boxhammer V (2013). Clin. Plasma Med..

[5] Hoffmann C, Berganza C, Zhang J (2013). Med. Gas Res..

[6] Stratmann B (2020). JAMA Netw. Open.

[7] Mirpour S, Fathollah S, Mansouri P, Larijani B, Ghoranneviss M, Mohajeri Tehrani M, Amini M R (2020). Sci. Rep..

[8] Lerouge S, Wertheimer M R, Yahia L (2001). Plasmas Polym..

[9] Moisan M, Barbeau J, Crevier M C, Pelletier J, Philip N, Saoudi B (2002). Pure Appl. Chem..

[10] Abbas M (2019). J. Hosp. Infect..

[11] O’Brien W J, Gupta K, Itani K M F (2020). JAMA Surg..

[12] Dumville J C, McFarlane E, Edwards P, Lipp A, Holmes A (2013). Cochrane Database Syst. Rev..

[13] Lange-Asschenfeldt B, Marenbach D, Lang C, Patzelt A, Ulrich M, Maltusch A, Terhorst D, Stockfleth E, Sterry W, Lademann J (2011). Skin Pharmacol. Physiol..

[14] Ulmer M, Lademann J, Patzelt A, Knorr F, Kramer A, Koburger T, Assadian O, Daeschlein G, Lange-Asschenfeldt B (2014). Skin Pharmacol. Physiol..

[15] Tendero C, Tixier C, Tristant P, Desmaison J, Leprince P (2006). Spectrochim. Acta B.

[16] Laroussi M, Lu X, Keidar M (2017). J. Appl. Phys..

[17] Hwang J H, Lee H Y, Chung K B, Lee H J, Kim J, Song K, Kim D Y (2021). Sci. Rep..

[18] Laurita R, Miserocchi A, Ghetti M, Gherardi M, Stancampiano A, Purpura V, Melandri D, Minghetti P, Bondioli E, Colombo V (2017). IEEE Trans. Radiat. Plasma Med. Sci..

[19] Lademann O, Kramer A, Richter H, Patzelt A, Meinke M C, Czaika V, Weltmann K D, Hartmann B, Koch S (2011). Skin Pharmacol. Physiol..

[20] Lademann O, Kramer A, Richter H, Patzelt A, Meinke M C, Roewert-Huber J, Czaika V, Weltmann K D, Hartmann B, Koch S (2011). Laser Phys. Lett..

[21] Yang F C, Zhang Y, Rheinstädter M C (2014). PeerJ.

[22] Otberg N, Richter H, Schaefer H, Blume-Peytavi U, Sterry W, Lademann J (2004). J. Invest. Dermatol..

[23] de Lacharrière O, Deloche C, Misciali C, Piraccini B M, Vincenzi C, Bastien P, Tardy I, Bernard B A, Tosti A (2001). Arch. Dermatol..

[24] Jimenez F, Izeta A, Poblet E (2011). Dermatol. Surg..

[25] Sun Q (2019). Nat. Commun..

[26] von Schuckmann L A, Hughes M C B, Ghiasvand R, Malt M, van der Pols J C, Beesley V L, Khosrotehrani K, Smithers B M, Green A C (2019). JAMA Dermatol..

[27] Norberg S A, Johnsen E, Kushner M J (2015). Plasma Sources Sci. Technol..

[28] Scharfetter D L, Gummel H K (1969). IEEE Trans. Electron Devices.

[29] Lietz A M, Kushner M J (2016). J. Phys. D: Appl. Phys..

[30] Babaeva N Y, Kushner M J (2010). J. Phys. D: Appl. Phys..

[31] Marzec E, Kubisz L (1997). Int. J. Biol. Macromol..

[32] Judée F, Dufour T (2019). J. Phys. D: Appl. Phys..

[33] Stancampiano A, Chung T H, Dozias S, Pouvesle J M, Mir L M, Robert E (2020). IEEE Trans. Radiat. Plasma Med. Sci..

[34] Chang Z S, Zhang G J, Shao X J, Zhang Z H (2012). Phys. Plasmas.

[35] Keidar M, Shashurin A, Volotskova O, Stepp M A, Srinivasan P, Sandler A, Trink B (2013). Phys. Plasmas.

[36] Norberg S A, Johnsen E, Kushner M J (2015). J. Appl. Phys..

[37] Darny T, Pouvesle J, Puech V, Douat C, Dozias S, Robert E (2017). Plasma Sources Sci. Technol..

[38] Lu X, Laroussi M, Puech V (2012). Plasma Sources Sci. Technol..

[39] Lloyd D P (1959). Nat. Physiol..

[40] Teschner T, Bansemer R, Weltmann K-D, Gerling T (2019). Plasma.

[41] Slikboer E, Viegas P, Bonaventura Z, Garcia-Caurel E, Sobota A, Bourdon A, Guaitella O (2019). Plasma Sources Sci. Technol..

[42] Babaeva N Y, Naidis G V, Tereshonok D V, Zhang C, Huang B, Shao T (2021). Plasma Sources Sci. Technol..

[43] Qi J, Garza L A (2014). Cold Spring Harb. Perspect. Med..

[44] Babossalam S, Abdollahimajd F, Aghighi M, Mahdikia H, Shokri B, Dilmaghanian A, Toossi P (2020). Arch. Dermatol. Res..

[45] Lee M, Kossard S, Wilkinson B, Doyle J A (1995). Aust. J. Dermatol..

